# Effects of brisk walking and Tai Chi interventions on sleep quality in university students with insomnia

**DOI:** 10.3389/fpubh.2025.1732579

**Published:** 2026-01-13

**Authors:** Xiaoyun Zhou, Feiyang Li, Ning Feng, Yuzhu Teng, Qianchun Yu

**Affiliations:** 1 Department of Social Medicine and Health Service Management, School of Health Management, Anhui Medical University, Hefei, Anhui, China; 2 Office of Academic Affairs, The First Affiliated Hospital of University of Science and Technology of China, Anhui Provincial Hospital, Hefei, Anhui, China; 3 Sports Human Science Research Center, School of Humanistic Medicine, Anhui Medical University, Hefei, Anhui, China; 4 Department of Physical Education, School of Humanistic Medicine, Anhui Medical University, Hefei, Anhui, China

**Keywords:** Tai Chi, Nordic walking, sleep quality, intervention, college student

## Abstract

**Objective:**

This study aimed to comparatively evaluate the interventional effects of two distinct modes of physical activity—brisk walking and Tai Chi—on subjective sleep quality among university students.

**Methods:**

The study was conducted between December 2023 and June 2024 at an undergraduate institution in Anhui Province. A total of 75 university students with a Pittsburgh Sleep Quality Index (PSQI) score >5 (indicative of sleep disturbance) were enrolled and randomly allocated in a 1:1:1 ratio to a Tai Chi group, a brisk walking group, or a control group. Over a 24-week period, the Tai Chi and brisk walking groups performed 60-min sessions three times per week, while the control group received no intervention and maintained their usual lifestyle and academic activities. Sleep quality was assessed using the PSQI before and after the intervention.

**Results:**

The mean age of the final analyzed sample was 18.77 ± 0.73 years. Following the 24-week intervention, statistically significant differences in PSQI total scores were observed among the three groups (*P* < 0.001). Both the Tai Chi and brisk walking groups exhibited significant reductions in PSQI scores following the intervention (Tai Chi group: 7.20 ± 1.63 vs. 4.52 ± 1.92; brisk walking group: 6.88 ± 1.65 vs. 4.42 ± 1.67; both *P* < 0.001). Within the Tai Chi group, significant improvements were noted in the subdomains of sleep duration, sleep efficiency, and daytime dysfunction (all *P* < 0.05). Similarly, the brisk walking group showed marked improvements in sleep quality, sleep duration, sleep efficiency, and daytime dysfunction (all *P* < 0.05). Furthermore, multiple linear regression analysis revealed that, compared to the control group, both the Tai Chi group (B = −1.889, *P* < 0.001) and the brisk walking group (B = −1.992, *P* < 0.001) were significantly associated with reductions in PSQI total scores, indicating that both exercise modalities effectively improved sleep quality.

**Conclusion:**

Both Tai Chi and brisk walking are associated with significant reductions in global PSQI scores among university students with insomnia, indicating their potential as effective non-pharmacological interventions for improving sleep quality in this population.

## 1 Introduction

Sleep is a vital physiological process essential for sustaining life. High-quality sleep, which requires adequate duration, plays a crucial role in regulating a wide range of metabolic and physiological functions (1). Research indicates that fluctuations in brainwave activity during sleep contribute to relaxation, memory consolidation, learning enhancement, critical thinking, and decision-making abilities, as well as improved academic performance (2). As a key component of the national youth system, university students undergo a critical developmental transition from secondary to tertiary education, during which they often encounter multiple challenges—including environmental adjustment, academic pressure, and financial burdens—that may substantially compromise their sleep quality (3). It has been reported that the prevalence of sleep disorders among Chinese university students is as high as 25.7% (4). Similarly, a study conducted in Italy involving 1,674 university students found that 54.6% of them experienced poor sleep quality (5).

Current frequently employed treatments for sleep disorders include cognitive behavioral therapy, pharmacotherapy, physical therapy, and exercise interventions (6). Among these, physical exercise has garnered significant attention as a safe, effective, and easily implementable approach to improving health outcomes (7). Studies have indicated that engagement in physical activity can facilitate better sleep, not only reducing sleep latency but also enhancing the overall sleep experience (8, 9). Common forms of exercise include aerobic activities, resistance training, and mind-body exercises. It should be noted, however, that although activities such as running can improve sleep effectively, they impose considerable stress on the knee joints and may be unsuitable for students with high body mass or pre-existing joint discomfort (10). Tai Chi, which integrates movement, breath control, and meditation, is classified as a light-to-moderate intensity physical activity with a metabolic equivalent ranging between 2.5 and 6.5 METs. It is characterized by low risk of injury, minimal cost, and flexible spatial requirements (11, 12). As a representative discipline within the physical education curricula of many universities, it offers a practical and sustainable option for student populations. Martial arts training is recognized not only for enhancing physical fitness among university students but also for contributing to psychological wellbeing (13, 14). As a traditional Chinese martial art, Tai Chi has long been utilized both domestically and internationally as a means of training and promoting health (13, 14). Meanwhile, brisk walking—another accessible form of aerobic exercise—falls within the moderate-intensity domain and offers distinct advantages such as simplicity, low joint impact, and suitability for the general healthy population (15). Previous research indicates that both aerobic and mind-body exercises can improve sleep quality. Given the potential of these two distinct modalities in sleep promotion, a comparative investigation into their interventional effects on sleep quality is of considerable scientific and practical significance (16, 17).

To ensure the scientific validity of the intervention protocol, the exercise prescription in this study was formulated by synthesizing existing evidence on physical activity for sleep improvement. Regarding methodological parameters, Tai Chi, as a mind-body exercise, is recommended for sleep enhancement with a regimen of 60-minute sessions conducted 2–7 times per week at light-to-moderate intensity (18). Brisk walking, as a moderate-intensity aerobic activity, should be performed at an intensity corresponding to 60–80% of maximum heart rate (19), with cumulative weekly durations of at least 150 min of moderate-intensity walking shown to improve sleep (20). Furthermore, research focusing on university students indicates that an exercise dose of at least three sessions per week, each lasting 30–60 minutes, confers positive effects on both physical and mental health (21). Consequently, an intervention protocol that aligns with these evidence-based benchmarks for sleep improvement while remaining feasible for long-term adherence among university students should incorporate multiple weekly sessions of approximately one-hour duration at moderate intensity. Accordingly, the present study adopted a standardized intervention schedule consisting of three 60-minute sessions per week over a 24-week period. This study was conducted to investigate the effects of two distinct modes of physical activity—brisk walking and Tai Chi—on subjective sleep quality in university students. Through comparative analysis, the findings are anticipated to contribute to the development of more scientifically grounded and effective sleep improvement strategies for this demographic.

## 2 Materials and methods

### 2.1 Study design

A randomized, three-arm, parallel controlled trial was conducted among undergraduate students with poor sleep quality (PSQI > 5) (22). The study was carried out between December 2023 and June 2024. Allocation concealment was achieved using computer-generated random number sequences prepared by researchers not involved in participant recruitment or outcome assessment, with assignments placed in sealed, opaque envelopes. Using this concealed allocation method, participants were randomly assigned to a Tai Chi group, a brisk walking group, or a control group. Participants in the Tai Chi and brisk walking groups underwent a 24-week supervised intervention program consisting of structured Tai Chi or brisk walking sessions, respectively. The control group received no intervention and was instructed to maintain their usual lifestyle and academic activities. Post-intervention data were collected by trained assessors upon completion of the 24-week period. The study design was implemented after receiving approval from the Ethics Committee of Anhui Medical University (Approval No.: 82230093).

### 2.2 Sample size calculation

The sample size was estimated using G^*^Power software (version 3.1). Based on previous research, a standardized mean difference (SMD) of 0.87 was assumed for the effect of exercise on sleep quality improvement. With a significance level (α) of 0.05, statistical power (1–β) of 0.8, and an allocation ratio of 1:1:1 among the Tai Chi group, brisk walking group, and control group, the calculated total sample size was 63 participants, with 21 individuals per group. To account for potential attrition and non-compliance during the trial, the final sample size was increased to 75 participants, resulting in 25 participants per group (23).

### 2.3 Participants

Participants were recruited from a university in Anhui Province, China. Undergraduate students with a PSQI score >5 were eligible for inclusion. Through campus-wide advertisements, 75 students with poor sleep quality were enrolled based on voluntary participation. Exclusion criteria included: (a) diagnosis of secondary insomnia; (b) experience of major life events within the past month (e.g., bereavement, romantic breakup); (c) engagement in regular exercise habits as assessed by the Physical Activity Rating Scale; (d) presence of severe cardiovascular, hematological, or psychiatric diseases, or any contraindications to physical activity; or (e) unwillingness to cooperate with study procedures. All participants provided written informed consent prior to enrollment.

Baseline data were collected prior to randomization. In addition to demographic information including age, gender, body mass index, residential background, parental education level, and household monthly income, participants' health behaviors and related experiences were assessed using a structured questionnaire, covering smoking status, alcohol consumption, romantic relationship experiences, and hospitalization history. The participating institution is a full-time residential university. According to its administrative regulations, all undergraduate students are required to reside in on-campus dormitories. Using a randomization procedure, participants were allocated to a Tai Chi group, a brisk walking group, or a control group for a 24-week intervention. All participants were instructed to maintain their habitual dietary intake, daily activity patterns, and medication regimens throughout the study period. Sleep-related metrics—including overall sleep quality, sleep latency, total sleep duration, sleep efficiency, sleep disturbances, use of hypnotic medications, and daytime dysfunction—were assessed using the PSQI both at baseline and following the completion of the 24-week intervention.

During the 24-week intervention period, one participant in the brisk walking group failed to complete the training protocol, and three participants in the control group withdrew from the study. Consequently, 25 participants from the Tai Chi group, 24 from the brisk walking group, and 22 from the control group were included in the subsequent analyses. In total, 71 participants (94.6%) completed the exercise program according to their group assignment.

### 2.4 Intervention protocol

The 24-form Tai Chi and moderate-intensity brisk walking were selected as intervention modalities in this study, primarily due to their established suitability for the university student population. University students frequently exhibit insufficient physical activity levels, with light-to-moderate intensity exercise recognized as an evidence-based approach to improving health outcomes (7, 24). Brisk walking, characterized by its low barrier to participation and minimal joint impact, is particularly well-suited as an introductory physical activity for insufficiently active students (15). Concurrently, studies indicate that Tai Chi, owing to its cultural congruence and integrated mind-body mechanism, represents an effective health intervention for this demographic (25).

During the 24-week intervention period, participants in the Tai Chi group and the brisk walking group engaged in their respective exercises three times per week, with each session lasting 60 min. Training sessions for both groups were scheduled on Tuesday, Thursday, and Saturday afternoons or evenings. The Tai Chi group followed a structured program based on the standardized 24-form Tai Chi routine. Each session began with a 5-min warm-up, consisting of preparatory movements for the shoulders, arms, legs, and waist. This was followed by 50 min of guided Tai Chi practice, concluding with a 5-min period dedicated to breathing regulation and relaxation exercises. In terms of progression, the first four weeks focused on mastering fundamental movements (e.g., hand positions, techniques, and leg methods), while the subsequent 20 weeks were devoted to systematic practice of the complete 24-form Tai Chi sequence. The brisk walking group performed exercises at a moderate intensity, with target heart rates maintained within 60%−80% of each participant's maximum heart rate (19). Each session commenced with a 3-minute warm-up, followed by a gradual increase in walking speed over 3–4 min until the target heart rate zone was achieved and stabilized. Participants then engaged in 50 min of sustained brisk walking, ending with a 3-min cool-down period. Throughout all sessions, heart rate monitoring devices were used to ensure adherence to the prescribed intensity range.

All sessions were conducted in designated venues under the guidance of experienced instructors. In contrast, participants in the control group did not receive any supervised physical activity and were instructed to maintain their habitual daily activity levels throughout the intervention period.

### 2.5 Outcome measures

Sleep quality was assessed using the Pittsburgh Sleep Quality Index (PSQI), a self-report instrument originally developed by Dr. Buysse and colleagues in 1989 that is widely utilized in the field of sleep medicine to evaluate an individual's subjective sleep quality over the preceding month. The PSQI comprises 19 self-rated items, which are subsequently grouped into seven components: subjective sleep quality, sleep latency, sleep duration, habitual sleep efficiency, sleep disturbances, use of sleeping medication, and daytime dysfunction. Each component is scored on a scale from 0 to 3, and the sum of the seven component scores yields the global PSQI score, ranging from 0 to 21. Higher total scores indicate poorer perceived sleep quality. Based on established cut-off values for this instrument, a total score greater than 5 is defined as “poor sleep quality,” with reported diagnostic sensitivity and specificity of 98% and 55%, respectively (22).

### 2.6 Statistical analysis

Data analysis was performed using SPSS Statistics (Version 26.0). Continuous variables conforming to a normal distribution are presented as mean ± standard deviation. Inter-group comparisons were conducted using one-way analysis of variance (ANOVA), while within-group pre-post comparisons were performed using paired-sample *t*-tests. Categorical data are summarized as frequencies and percentages, with inter-group comparisons carried out using chi-square tests or Fisher's exact test, as appropriate. To evaluate the independent effect of the interventions, a multiple linear regression model was constructed, with the post-intervention PSQI total score as the dependent variable and group assignment (represented using dummy variables) as the independent variable. All hypothesis tests were two-tailed, with the significance level (α) set at 0.05.

## 3 Results

### 3.1 Comparison of baseline characteristics among the three groups

Baseline data from 71 students were included in the analysis. No statistically significant differences were observed among the Tai Chi group, control group, and brisk walking group in terms of age, body mass index (BMI), gender, residential background, monthly household income per capita, parental education level, smoking or alcohol consumption status, experience of romantic breakup, or history of hospitalization (all *P* > 0.05). Detailed results are presented in Table 1.

**Table 1:** Descriptive statistics and comparative analysis of demographic characteristics at baseline.

**Variable**		**Tai Chi (*n* = 25)**	**Control (*n* = 22)**	**Brisk walking (*n* = 24)**	***χ^2^/F***	***P***
Age, years		19.24 ± 0.66	18.86 ± 0.64	18.96 ± 0.69	2.071	0.134
BMI, kg/m^2^		21.36 ± 2.29	20.73 ± 3.00	21.44 ± 3.33	0.414	0.663
Height, cm		166.16 ± 8.08	165.16 ± 7.32	168.41 ± 6.11	1.186	0.312
Weight, kg		59.05 ± 8.27	58.67 ± 10.91	58.61 ± 7.53	0.017	0.984
Gender, *n* (%)					1.826	0.401
	Male	4 (22.2)	7 (38.9)	7 (38.9)		
	Female	21 (39.6)	15 (28.3)	17 (32.1)		
Residential area, *n* (%)					4.457	0.615
	Urban	7 (28.0)	6 (27.3)	7 (29.2)		
	Suburban	3 (12.0)	1 (4.5)	2 (8.3)		
	County	7 (28.0)	4 (18.2)	9 (37.5)		
	Rural	8 (32.0)	11 (50.0)	6 (25.0)		
Monthly household income per capita, *n* (%)					7.770	0.255
	< 2,000 CNY	2 (8.0)	2 (9.1)	1 (4.2)		
	2,001–5,000 CNY	16 (64.0)	13 (59.1)	10 (41.7)		
	5,001–10,000 CNY	5 (20.0)	7 (31.8)	8 (33.3)		
	>10,000 CNY	2 (8.0)	0 (0)	5 (20.8)		
Paternal education level, *n* (%)					5.166	0.523
	Primary or below	3 (12.0)	3 (13.6)	3 (12.5)		
	Junior	16 (64.0)	9 (40.9)	9 (37.5)		
	Senior high or secondary	3 (12.0)	7 (31.8)	7 (29.2)		
	College or above	3 (12.0)	3 (13.6)	5 (20.8)		
Maternal education level, *n* (%)					3.603	0.730
	Primary or below	9 (36.0)	7 (31.8)	7 (29.2)		
	Junior	8 (32.0)	6 (27.3)	11 (45.8)		
	Senior high or secondary	5 (20.0)	7 (31.8)	3 (12.5)		
	College or above	3 (12.0)	2 (9.1)	3 (12.5)		
Smoking, *n* (%)					1.986	0.366
	Current smoker	0	0	0		
	Former smoker (abstinent ≥3 months)	0	0	1 (4.2)		
	Never smoked	25 (100)	22 (100)	23 (59.8)		
Alcohol use, *n* (%)					3.406	0.182
	Non-drinker	16 (64.0)	19 (86.4)	19 (79.2)		
	Occasional drinker (1–2 times/month)	9 (36.0)	3 (13.6)	5 (20.8)		
	Frequent drinker (≥1 time/week)	0	0	0		
Romantic breakup, *n* (%)					1.357	0.507
	Yes	3 (12.0)	3 (13.6)	1 (4.2)		
	No	22 (88)	19 (86.4)	23 (95.8)		
Hospitalization history, *n* (%)					0.925	0.630
	Yes	1 (4.0)	0 (0)	1 (4.2)		
	No	24 (96)	22 (100)	23 (95.8)		

Continuous variables are presented as mean ± standard deviation and were compared using analysis of variance (F-statistic). Categorical variables are presented as number (percentage) and were compared using the chi-square test (χ^2^statistic).

### 3.2 Comparison of PSQI total scores among the three groups before and after intervention

As summarized in Table 2, no statistically significant differences were observed in baseline PSQI total scores across the three groups (F = 0.276, *P* = 0.760), establishing baseline comparability. Following the 24-week intervention, a statistically significant divergence in PSQI scores was detected among the groups (F = 9.571, *P* < 0.001). Furthermore, paired-samples *t*-tests in Table 2 demonstrated that both the Tai Chi group and the brisk walking group exhibited statistically significant reductions in PSQI total scores post-intervention compared to their respective baseline values (both *P* < 0.001). In contrast, the control group showed no statistically significant change in PSQI scores between pre- and post-intervention assessments (*P* = 0.147).

**Table 2:** Comparison of total PSQI Scores among the three groups before and after the intervention.

**Group**	***n***	**Pre-intervention PSQI**	**Post-intervention PSQI**
Tai Chi	25	7.20 ± 1.63	4.52 ± 1.92^*^
Brisk walking	24	6.88 ± 1.65	4.42 ± 1.67^*^
Control	22	7.00 ± 1.13	6.41 ± 1.56
*F*		0.276	9.571
*P*		0.760	**< 0.001**

Values are presented as mean ± standard deviation. ^*^denotes a statistically significant within-group difference compared to pre-intervention (*P* < 0.001). Bold values indicate statistically significant differences in the post-intervention comparison among the three groups (*P* < 0.001).

### 3.3 Comparison of PSQI subdomain scores among the three groups before and after intervention

Following the intervention, significant reductions were observed in the Tai Chi group regarding scores for sleep duration, sleep efficiency, and daytime dysfunction compared to baseline measurements (all *P* < 0.05). Similarly, the brisk walking group exhibited statistically significant improvements in the subdomains of sleep quality, sleep duration, sleep efficiency, and daytime dysfunction after the intervention (all *P* < 0.05). In contrast, no significant differences were detected in any of the PSQI subdomain scores within the control group between pre- and post-intervention assessments. Detailed results are presented in Table 3.

**Table 3:** Comparison of PSQI component scores before and after intervention among the three groups.

**Group**	***n***	**Component**	***Z***	***P***
Tai Chi	25	Sleep Quality	−0.378	0.705
		Sleep Latency	−1.795	0.073
		Sleep Duration	−3.755	**0.001**
		Sleep Efficiency	−3.419	**0.001**
		Sleep Disturbance	−1.134	0.257
		Use of Sleep Medication	0.000	1.000
		Daytime Dysfunction	−2.336	**0.019**
Brisk Walking	24	Sleep Quality	−3.162	**0.002**
		Sleep Latency	−1.500	0.134
		Sleep Duration	−3.234	**0.001**
		Sleep Efficiency	−2.448	**0.014**
		Sleep Disturbance	−0.707	0.480
		Use of Sleep Medication	0.000	1.000
		Daytime Dysfunction	−2.595	**0.009**
Control	22	Sleep Quality	−1.000	0.317
		Sleep Latency	−1.387	0.166
		Sleep Duration	−1.508	0.132
		Sleep Efficiency	−0.443	0.658
		Sleep Disturbance	−0.333	0.739
		Use of Sleep Medication	−1.000	0.317
		Daytime Dysfunction	−1.182	0.237

Bold P-values indicate a statistically significant within-group difference before and after the intervention (*P* < 0.05).

### 3.4 Multiple linear regression analysis of the effects of exercise interventions on sleep quality

To examine the independent effects of different exercise modalities on sleep quality, a multiple linear regression analysis was performed with the global PSQI score as the dependent variable. Group assignment served as the independent variable, represented by two dummy variables (Tai Chi group and brisk walking group), using the control group as the reference category. The results indicated that the overall model fit was statistically significant [F_(2, 68)_ = 9.571, ^*^p^*^ < 0.001], with an adjusted R^2^ of 0.197, indicating that the two exercise intervention variables collectively explained 19.7% of the variance in PSQI scores. Compared to the control group, the Tai Chi group demonstrated a significantly lower PSQI total score, with an average reduction of 1.889 points (*B* = −1.889, *P* < 0.001). Similarly, the brisk walking group showed a significantly lower PSQI total score relative to the control group, with an average reduction of 1.992 points (*B* = −1.992, *P* < 0.001), as detailed in Table 4.

**Table 4:** Multiple linear regression analysis of the effects of different exercise interventions on PSQI total scores.

**Variable**	**Unstandardized coefficient *B***	**Standard error**	**Standardized coefficient β**	***t-*value**	***p-*value**
Constant	6.409	0.369	-	17.382	< 0.001
Tai Chi	−1.889	0.506	−0.471	−3.737	< 0.001
Brisk walking	−1.992	0.510	−0.492	−3.903	< 0.001

*R*^2^ = 0.220, Adjusted R^2^ = 0.197, *F* = 9.571, *p* < 0.001.

## 4 Discussion

This 24-week randomized controlled trial (RCT) systematically compared the effects of two distinct exercise modalities—brisk walking and Tai Chi—on sleep quality among university students with insomnia. The results demonstrate that both interventions significantly improved overall sleep quality, with positive outcomes observed across multiple PSQI subdomains.

The primary findings align with previous research, such as the study by Siu PM et al., which reported beneficial effects of Tai Chi on sleep quality (26). Consistent with earlier research, our study confirms that Tai Chi not only enhances global sleep quality but also leads to significant improvements in specific dimensions including sleep duration, sleep efficiency, and daytime dysfunction (27). Moreover, brisk walking was notably effective in improving sleep quality, sleep efficiency, and daytime functioning. This observation is supported by existing literature indicating that moderate-intensity aerobic exercise can produce substantial improvements in both global PSQI scores and key subcomponents such as sleep efficiency and daytime dysfunction (28), thereby reinforcing the efficacy of brisk walking as a practical and accessible intervention for sleep enhancement.

Regarding the design of the intervention protocol, a systematic review indicates that exercise performed at a low frequency, short duration, and low-to-moderate intensity can effectively improve sleep quality, with particular emphasis on aerobic exercise and Tai Chi as especially beneficial modalities (29). Specifically, evidence-based Tai Chi protocols commonly associated with sleep improvement involve sessions of 60 min each, conducted 2 to 7 times per week over a period of 3 to 6 months (18). Similarly, regular moderate-intensity walking (such as accumulating 150 min per week) has also been shown to contribute to sleep enhancement (20). The intervention design in the present study aligns with the evidence-supported effective dosage window in terms of exercise type, session duration, and intervention period. This alignment ensures that the intervention dose falls within a known effective range, thereby providing a solid foundation for the scientific validity and comparability of the study outcomes.

Although the majority of the present findings are consistent with existing literature, certain discrepancies were observed regarding the relative efficacy of Tai Chi vs. brisk walking. This study revealed comparable improvements in PSQI total scores between the two exercise modalities, whereas the RCT controlled trial conducted by Takemura et al. suggested superior effects of Tai Chi over aerobic exercise (16). Several factors may account for this divergence.

First, differences in sample characteristics may have contributed to the discrepant outcomes. The current study enrolled university students with PSQI > 5 and a mean age of approximately 19 years, whereas Takemura et al. focused on middle-aged and older adults with insomnia. Age-related differences in physiological and psychological responses to exercise may influence the relative effectiveness of distinct movement modalities.

Second, variations in intervention protocols could explain the contrasting results. The brisk walking group in our study participated in supervised, group-based sessions—a structured design likely to enhance adherence and optimize exercise intensity. In contrast, several studies reporting superior benefits of Tai Chi often implemented unsupervised or home-based aerobic training protocols, which may lead to insufficient compliance and suboptimal exercise dosage (16).

Third, the sensitivity of assessment tools may also affect outcome comparisons. While this study utilized the PSQI as the primary endpoint, some investigations have observed that Tai Chi may demonstrate more pronounced advantages when evaluated using objective sleep measures (30). Differences in measurement approaches could thus influence the comparative conclusions regarding intervention efficacy.

At the mechanistic level, both Tai Chi and brisk walking may contribute to improved sleep quality through the modulation of the neuro-immune-endocrine axis. Specifically, these two modalities share a common pathway in promoting the synthesis and release of serotonin (5-HT), which is known to enhance sleep stability (31, 32). Furthermore, brisk walking, as a representative moderate-intensity aerobic exercise, may facilitate autonomic nervous system balance. Coupled with exercise-induced thermoregulatory changes, it could further promote the occurrence of slow-wave sleep, thereby reinforcing the regulation of the sleep-wake cycle across multiple dimensions (33, 34).

This study possesses several notable strengths. First, the adoption of a three-arm, parallel, RCT design, complemented by concealed allocation and baseline equivalence testing, effectively minimized selection bias and enhanced the internal validity of the findings. Second, the 24-week intervention period was substantially longer than that of most comparable studies. Combined with a supervised, group-based training model, this approach ensured consistency in exercise frequency and duration, as well as high participant adherence, thereby providing high-quality evidence regarding the long-term effects of physical activity on sleep improvement. Furthermore, the joint analysis of both global and subdomain PSQI scores not only confirmed overall sleep enhancement but also elucidated the differential effects of Tai Chi and brisk walking across specific sleep dimensions. These nuanced findings offer an empirical basis for developing personalized exercise prescriptions. Finally, by directly comparing the sleep-related benefits of Tai Chi and brisk walking within a university student population, this study addresses a notable gap in exercise intervention research among young adults. The results demonstrate comparable efficacy between the two modalities despite distinct profiles of subdomain improvements, thereby affording flexible and evidence-based options for sleep health promotion in higher education settings.

This study has several limitations that should be acknowledged. First, the participants were recruited from a single university, and the sample had a relatively high proportion of female students, which may limit the generalizability of the findings. Future studies should aim to include larger and more geographically diverse populations to enhance external validity. Second, the absence of physiological biomarkers—such as neuroendocrine or inflammatory markers—prevents a deeper exploration of the biological mechanisms through which exercise improves sleep. Incorporating such measures in subsequent research would provide valuable insights into the underlying pathways. Third, reliance on the self-reported PSQI questionnaire, without objective sleep measurements such as polysomnography or actigraphy, may not fully capture the effects of exercise on sleep architecture. Future investigations would benefit from integrating multidimensional sleep assessments to obtain a more comprehensive understanding of intervention effects.

It should also be noted that although all participants engaged in a routine physical education class lasting 40 min per week, this activity differed fundamentally from the structured intervention implemented in the present study in terms of content, intensity, and frequency. Moreover, exposure to this routine class was consistent across the three groups, thereby ensuring that between-group comparisons were not systematically confounded by differential background levels of physical activity.

## 5 Conclusion

This 24-week RCT demonstrates that both Tai Chi and brisk walking significantly improve sleep quality among university students with insomnia, as evidenced by reductions in global PSQI scores and specific enhancements in domains such as sleep efficiency and daytime dysfunction. While the overall efficacy of the two exercise modalities was comparable, each exhibited distinct advantages across different sleep dimensions. Future research should focus on elucidating the underlying mechanisms of exercise-induced sleep improvement, refining personalized exercise prescriptions, and evaluating the long-term benefits of various intervention strategies to more effectively address the prevalent issue of sleep disturbances in the student population.

## Data Availability

The raw data supporting the conclusions of this article will be made available by the authors, without undue reservation.
